# Impact of plant-based diets and associations with health, lifestyle and healthcare utilisation: a population-based survey study

**DOI:** 10.1017/S1368980025100669

**Published:** 2025-07-17

**Authors:** Natalia Echiburu, Maria Antonieta Also-Fontanet, Antoni Sisó-Almirall, Luis González-de Paz

**Affiliations:** 1 Consorci d’Atenció Primària de Salut Barcelona Esquerra (CAPSBE), Barcelona, Spain; 2 Primary Healthcare Transversal Research Group, Institut d’Investigacions Biomèdiques, August Pi i Sunyer (IDIBAPS), Barcelona, Spain; 3 Department of Medicine, Universitat de Barcelona (UB), Barcelona, Spain; 4 Department of Public Health, Mental Health and Mother and Child Health, Universitat de Barcelona (UB), Barcelona, Spain

**Keywords:** Plant-based diet, Vegan, Vegetarian, Lifestyle, Chronic diseases, Survey design

## Abstract

**Objective:**

To determine the prevalence and characteristics of plant-based patterns in the Spanish population and assess their potential impact on individuals with similar socio-demographic backgrounds.

**Design:**

We estimated vegetarian and vegan individuals’ national and regional prevalence and analysed their socio-demographic characteristics and weekly dietary intake patterns. Individuals with a plant-based dietary pattern were matched to a control group (1:4) with similar socio-demographic characteristics. Associations with the prevalence risk of common chronic diseases, self-reported health status, lifestyle and healthcare use were analysed with unadjusted and adjusted logistic regression models.

**Setting:**

A population-based survey of individuals residing in Spain.

**Participants:**

Data from 22 072 participants were examined.

**Results:**

The prevalence of plant-based diets was 5·62‰ (95 % CI: 4·33, 7·28), and adherents were female (68·6 %), single (62·3 %) and university-educated 41·8 %) (*P* < 0·001). They reported higher rates of ‘never’ consuming snacks (50 % *v*. 35 %), fast food (58 % *v*. 36 %) and sweets (33 % *v*. 14 %). Lifestyle factors did not differ between the plant-based and omnivorous groups; however, adherence to plant-based diets was associated with a prevalence risk of depressive symptoms (OR 2·58, 95 % CI: 1·00, 6·65), stroke (OR 7·08, 95 % CI: 1·27, 39·46) and increased consultations for mental health and complementary medicine (OR 3·21, 95 % CI: 1·38, 7·43).

**Conclusions:**

Plant-based diets are uncommon and are associated with specific socio-demographic profiles, particularly sex. When comparing individuals with similar socio-demographic characteristics, individuals with plant-based diets and omnivores had similar lifestyles. Addressing patient concerns regarding diet and personal well-being might prioritise healthy behaviours over specific dietary patterns.

Plant-based dietary patterns are increasingly accepted for ethical concerns and environmental sustainability^(1)^, and when appropriately planned, can provide adequate nutrition across all stages of life^(2)^. While some vegetarian diets may include some animal-derived products such as dairy, eggs and honey, both vegetarian and vegan diets exclude the intake of animal products such as meat, poultry and fish. Vegan diets further exclude all animal-based foods and by-products^(3)^. The prevalence of plant-based dietary patterns has been scientifically studied using health survey data. In Canada, a prevalence of 2·8 % was reported^(4)^, and in the USA, a prevalence of 4·0 %^(5)^. In the New Zealand health survey, a 2·04 % prevalence of vegetarians and a 0·74 % of vegans was reported^(6)^, and in Europe, studies reported prevalences <1 %^(7,8)^. However, studies using representative population surveys have not applied survey-weighting factors, which allows accurate prevalence estimation to correct for sample bias, adjust for imbalances and allow for generalisable results in underrepresented groups, such as plant-based individuals^(9)^.

Individuals with plant-based diets have been reported to have healthier habits like consuming less alcohol or not smoking^(10)^. Compared with omnivores, vegetarians and vegans often exhibit lower BMI, total cholesterol, LDL, TAG and blood glucose levels^(11,12)^. These reductions in risk factors are linked to a decreased likelihood of developing chronic diseases, including CVD, diabetes, obesity, chronic kidney failure and various cancers^(13)^. Predominantly, plant-based diets improve metabolic control in individuals with diabetes^(14)^ and metabolic syndrome, reducing the risk of conditions such as ischemic heart disease^(15)^, hypertension^(16)^, chronic kidney disease^(17)^, obesity^(18)^, fatty liver disease^(19)^ and cancer onset^(20,21)^. However, plant-based diets can be associated with nutrient deficiencies if not adequately performed. A strict plant-based diet is associated with increased susceptibility to experiencing bone fractures, osteoporosis and haemorrhagic stroke^(16)^. Despite evidence suggesting health advantages associated with plant-based adherence, comprehensive studies remain lacking in integrating individuals’ health status and lifestyle factors perspective (e.g. physical activity, alcohol drinking habit or tobacco consumption habit). Moreover, few studies have compared these individuals to nonvegetarians with similar social determinants of health (e.g. educational level, household income or marital status).

Comparing individuals with an omnivore diet and a plant-based dietary pattern has explored the physiological outcomes associated with plant-based diets; however, these studies often failed to account for lifestyle habits and socio-demographic characteristics. Therefore, this study aimed to (i) determine the prevalence of vegetarian and vegan plant-based diets in the Spanish population and (ii) assess the impact of these dietary patterns on perceived quality of life, lifestyle, prevalence risk of common diseases and healthcare service utilisation in individuals with similar socio-demographic backgrounds.

## Methods

### Study design

We conducted a two-phase study: first, we assessed the prevalence of individuals adhering to a plant-based diet (vegetarians and vegans) in a representative sample of Spanish adults living in their households. We also examined the socio-demographic characteristics of the plant-based and omnivore groups. In the second phase, we conducted a matched cross-sectional analysis with all plant-based diet individuals matched to a sample of participants with similar socio-demographic characteristics and an omnivorous diet to study the association of plant-based diets with perceived quality of life, lifestyle factors, diseases and healthcare service utilisation in the last year.

### Source of data and context

The European Health Interview Survey in Spain (EHIS), conducted by the Spanish Statistics Institute and the Ministry of Health, is a household survey conducted every 5 years that collects health-related information on the population residing in Spain aged ≥15 years old, using a standard European questionnaire^(22,23)^. The EHIS data are a source of information for countrywide statistics on health, morbidity, the extent of access to and use of healthcare services and the factors influencing health-related outcomes^(22)^. The EHIS collects a countrywide sample. It uses the family dwelling as the basic unit, gathering information voluntarily and anonymously. The sampling of the EHIS used a multistage clustering method with proportional random selection and independent samples from different Spanish regions. The survey was conducted in participants’ homes using face-to-face, computer-assisted personal interviews with trained interviewers. The methodology allows the delivery of long questionnaires, and the interviewer can assist in explaining the questions and demonstrating examples of how to complete the items. Data collected through this methodology are self-reported by the participants. The latest available data from 2020 included responses from 22 072 individuals. Further methodological details are available elsewhere^(24)^.

### Participants and matching criteria

We selected all individuals from the EHIS in Spain in the last available year (2020) with a complete form of a weekly FFQ included in the survey module on the dietary habits of the EHIS. It is a practical and standardised instrument that has been compared against a 7-day food diary and a seventy-four-item FFQ with strong reliability^(25)^. It asks for fourteen food groups with six answer categories (once or more times a day, 4–6 times a week, three times a week, once or twice a week, less than once a week and never). We considered vegetarian to those participants reporting ‘never’ consuming meat, fish or processed meat products (e.g. salami, sausages and hot dogs); vegans if answered ‘never’ on meat, fish, processed meat, dairy or eggs and omnivores if answered ‘never’ to meat, fish and processed meat groups. The control participants (omnivores) did not avoid meat or other animal-derived foods in their diet (omnivores), according to the FFQ.

### Study variables

Data on socio-demographic variables were collected on age (years), sex, living situation (living with a spouse, with cohabitating partner or living alone), marital status, completion of higher academic studies, household income and social class, categorised with the six-group classification adopted by the Spanish Society of Epidemiology, which is based on the occupation of the household reference person^(26)^ and health insurance coverage used.

The prevalence of chronic diseases was examined using the item ‘*Has a physician diagnosed any of the following diseases?’.* We used the Patient Health Questionnaire for depression symptoms. The Patient Health Questionnaire-8 is a valid instrument for monitoring depression and studying its prevalence^(27)^. Self-perceived health was studied with the EHIS question ‘*How is your health in general?’* reported as very good, good, fair, bad and very bad. The variables on lifestyle habits included tobacco consumption (non-smokers, smokers and ex-smokers and hazardous alcohol drinking, which were measured using the Standard Drink Units (SDU). In Spain, one fermented beverage (e.g. beer, wine) is set to one 1 SDU, and a distilled beverage (e.g. spirit, liquor) is set to two SDU^(28)^. We studied the weekly hazardous drinking (weekly alcohol intake >28 SBU in men or >17 SBU in women)^(29)^. Physical activity was measured according to the WHO enhancing physical activity recommendations (1): aerobic compliance (≥150 min of at least moderate-intensity aerobic physical activity per week), muscle-strengthening compliance (≥2 times per week), and aerobic and muscle-strengthening if both. Variables on the use of healthcare services included a reported consultation (yes/no) during the preceding year with a General Practitioner, a physician specialist (not a General Practitioner), a physiotherapist, a kinesiotherapist, a chiropractor or osteopath, a psychologist, psychotherapist or psychiatrist, a registered nurse or nurse practitioner and a complementary medicine practitioner (homeopathist, naturopath or acupuncturist). Consumption of medicines was studied using the following questions: (*During the past two weeks, have you used any medicines prescribed by a doctor*? (yes/no) and *have you used any medicines, herbal medicines or vitamins for the past two weeks?* (yes/no), we also examined the reported consumption of vitamins, minerals or tonics and naturopathic or homeopathic products.

### Statistical analysis

All participants were classified as vegetarians, vegans or omnivores according to their FFQ answers. We examined the frequency of weekly intake in the fourteen food groups. The prevalence of plant-based diets was computed using the whole EHIS sample, and population estimates with its 95 % CI were calculated using weighting factors for the entire Spanish population and 1eighteen Spanish regions. The socio-demographic characteristics of participants with plant-based and omnivore diets were described using central and frequency statistics, and differences between groups across socio-demographic characteristics were examined using ANOVA or χ^2^ tests.

In the second phase, the matching process of participants with a plant-based diet to a sample of participants with an omnivore diet based on socio-demographic factors that showed statistically significant differences in the first phase (*P* value < 0·05) was carried out using a propensity score analysis with the nearest neighbor criteria (Caliper = 0·08, *r* = 1:4). The matching method was used to reduce confounding bias and dependence by balancing the group of participants with omnivore and vegetarian diets in selected covariates. After matching, the final data were expected to be closer to a block-randomised design, and we examined the balance with the standardised mean differences of the propensity scores^(30,31)^.

The association of plant-based diets with the prevalence of chronic diseases, self-perceived general health (bad or very bad categories), lifestyle and risk factors and use of healthcare services and medicine consumption were calculated using logistic regression models in two steps: Initially, we computed logistic regression models with the plant-diet or omnivore diet as the response factor. Subsequently, we adjusted the association for the influence of residual differences and variance between groups by introducing the socio-demographic covariates from the first analysis into the models. The results are presented as OR and adjusted OR with their respective 95 % CI and *P* values. In all analyses, the level of statistical significance was set at α = 0·05. All analyses were performed using R version 4.2.2^(32)^.

## Results

**Figure 1.**
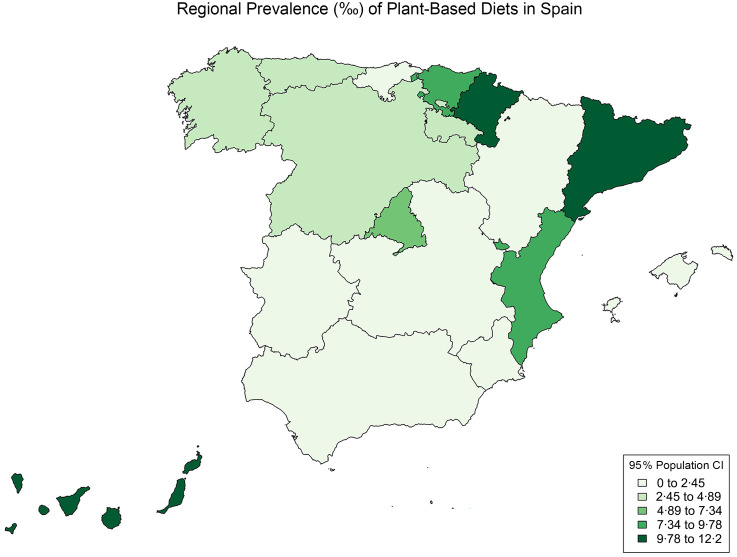
Population prevalence in Spanish regions.

We identified eighty-six individuals following a plant-based diet, with a population prevalence in Spain of 5·62‰ (95 % CI: 4·33, 7·28). Of these, eighteen reported consuming a vegan diet (prevalence 1·31‰, 95 % CI: 0·70, 2·20), and sixty-eight followed a vegetarian diet (prevalence 4·31‰, 95 % CI: 3·18, 5·69). Three regions had no vegetarians, and eight had no vegans. Due to the small sample sizes of the vegan and vegetarian groups, we combined both groups. The Iberian Peninsula’s northern and central Mediterranean regions had higher prevalences, ranging from 10 to 12·23, respectively, whereas the central and southern regions had lower rates, between 1·52 and 6·64. Figure 1 presents a map of prevalence distribution across Spanish regions, with detailed regional data in Tables A, B and C in the online appendix.

**Figure 2.**
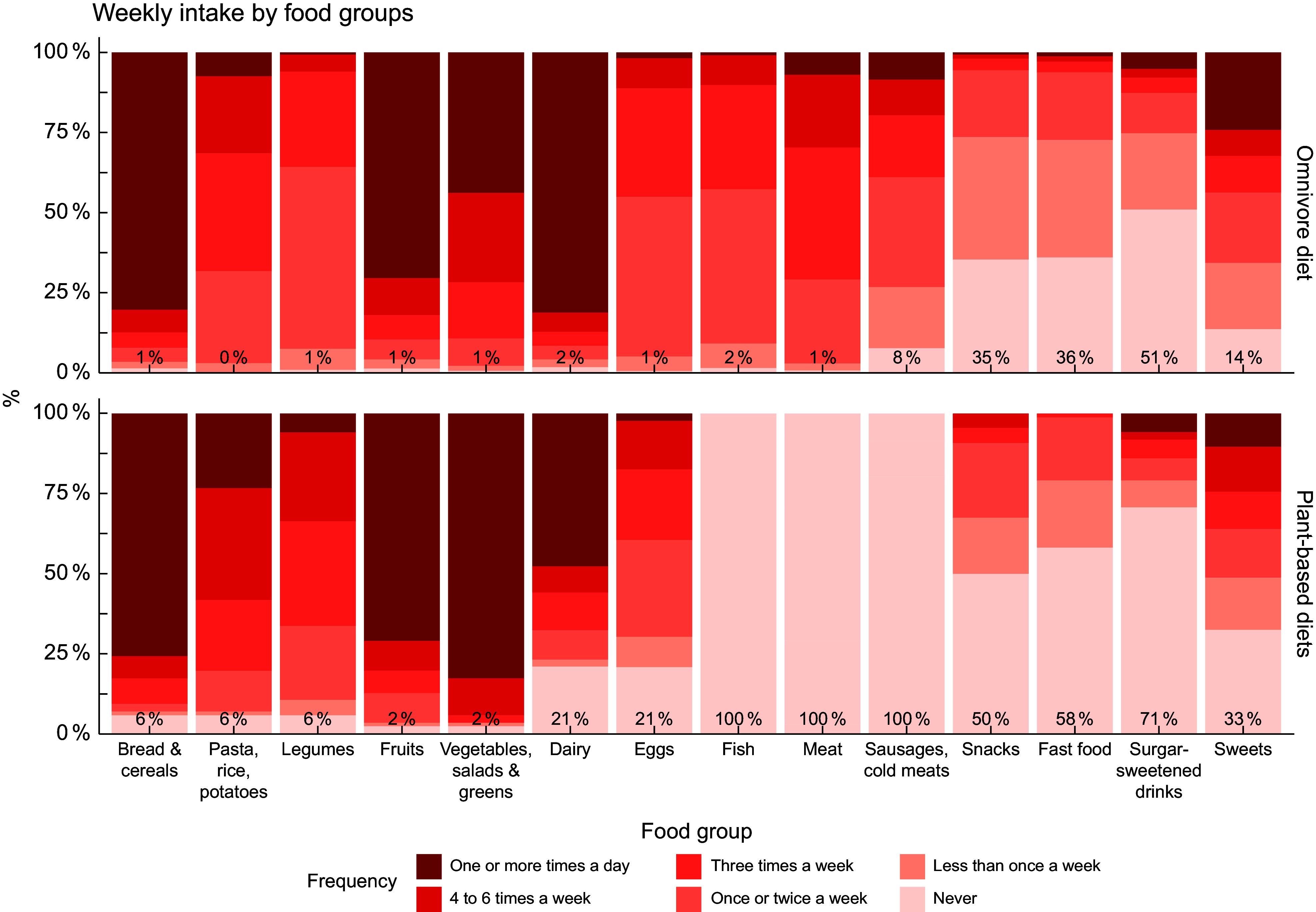
Weekly food intake by food groups, as reported by plant-based and omnivore participants.

Figure 2 presents a stacked bar chart illustrating weekly food intake, with color intensity indicating the intake frequency by food group. Darker shades represent higher weekly intake (e.g. daily consumption). Individuals adhering to a plant-based dietary pattern had higher proportions in the *never* category across all food groups, with differences in snacks (50 % *v*. 35 %), fast food (58 % *v*. 36 %) and sweets (33 % *v*. 14 %). Individuals following a plant-based dietary pattern reported higher intake frequencies of vegetables, salads and greens, with 82·6 % consuming them *once or more times per day* compared with 43·9 % of omnivores. Additionally, over two-thirds (66·2 %) of plant-based individuals consumed legumes three or more times per week, compared with 33·3 % of omnivores. In contrast, differences in the never consumption of staple plant-based foods – such as bread and cereals (6 % *v*. 1 %), pasta, rice or potatoes (6 % *v*. 0 %), and legumes (6 % *v*. 1 %) – were minimal, as both groups showed relatively high overall consumption levels.

The socio-demographic characteristics in Table 1 show that individuals with a plant-based diet were more likely to be women than those with an omnivore diet (69·08 % *v*. 52·80 %, *P* < 0·001), they were younger (mean age 48·42 *v*. 54·60 years, *P* < 0·001) and more often not living with a partner (70·93 % *v*. 49·19 %, *P* < 0·001), with a majority being single (60·35 % *v*. 27·04 %, *P* < 0·001). Additionally, individuals on a plant-based diet had higher educational attainment (university degree or higher: 41·86 % *v*. 19·29 %, *P* < 0·001). They also reported higher household incomes and social classes than those with an omnivorous diet (*P* < 0·05) and a higher proportion of healthy weight than omnivores (42·76 % *v*. 67·47 %, *P* < 0·001). The matching process of eighty-six individuals following a plant-based diet to 342 matched omnivores (1:4 ratio) reduced to almost 0 the standardised mean differences in age (deciles), sex, household living situation, marital status, educational attainment, household income, BMI and social class (Figure A in the online appendix). Additionally, we conducted a sensitivity analysis to assess whether BMI differed between the omnivorous and vegetarian groups when it was not included in the matching process, and we did not find a statistically significant effect. This result is presented in Figure B in the online appendix.

**Table 1.** Socio-demographic characteristics of individuals with plant-based diets and omnivores

Characteristics	Omnivores		Plant based		*P* value
	*n* 21 837		*n* 86		
	*n*	%	*n*	%	
Age (years)	54·59	18·99	42·35	17·76	<0·001
Sex (female)	11 550	52·89 %	59	68·60 %	0·005
Cohabitation in the household					<0·001
Living with a spouse	10 611	48·94 %	22	25·58 %	
Living with a cohabiting partner	396	1·83 %	3	3·49 %	
Not living with a partner	10 674	49·23 %	61	70·93 %	
Marital status					<0·001
Single	5887	27·04 %	53	62·35 %	
Married	11 204	51·45 %	22	25·88 %	
Widowed	2867	13·17 %	4	4·71 %	
Legally separated	646	2·97 %	3	3·53 %	
Divorced	1171	5·38 %	3	3·53 %	
Academic studies have ended					<0·001
Primary education	6267	28·70 %	9	10·47 %	
High school has not finished	5283	24·19 %	10	11·63 %	
High-school finished	2754	12·61 %	20	23·26 %	
Vocational and professional studies	3322	15·21 %	11	12·79 %	
University degree of higher	4211	19·28 %	36	41·86 %	
Household income					0·025
<€1100	6647	30·62 %	30	36·14 %	
€1100 to <€1650	4192	19·31 %	15	18·07 %	
€1650 – <€2300	3954	18·21 %	5	6·02 %	
€2300 – <€3800	4674	21·53 %	19	22·89 %	
≥€3800	2242	10·33 %	14	16·87 %	
Social class					0·006
I	2266	10·86 %	13	15·48 %	
II	1655	7·94 %	14	16·67 %	
III	4182	20·05 %	22	26·19 %	
IV	3022	14·49 %	8	9·52 %	
V	6870	32·94 %	19	22·62 %	
VI	2861	13·72 %	8	9·52 %	
Health insurance					0·087
Private service	2841	13·01 %	18	20·93 %	
Public Health System	18 928	86·68 %	68	79·07 %	
Others or does not have	68	0·31 %	0	0·00 %	
Size of the municipality of the residence					0·575
>500 000 inhabitants	2647	12·12 %	11	12·79 %	
Provincial capital	4611	21·12 %	16	18·60 %	
>100 000 inhabitants	1873	8·58 %	8	9·30 %	
50 000–100 000 inhabitants	1990	9·11 %	10	11·63 %	
20 000–50 000 inhabitants	3516	16·10 %	19	22·09 %	
10 000–20 000 inhabitants	2366	10·83 %	9	10·47 %	
<10 000 inhabitants	4834	22·14 %	13	15·12 %	
BMI					<0·001
Underweight	399	1·93 %	7	8·43 %	
Healthy weight	8853	42·76 %	56	67·47 %	
Overweight	8101	39·13 %	13	15·66 %	
Obese	3350	16·18 %	7	8·43 %	

The study on lifestyle factors, healthcare utilisation and medication use showed that individuals following plant-based diets were had a higher likelihood of visiting a mental health professional (adjusted OR: 3·21, 95 % CI: 1·38, 7·43) and were more likely to consult complementary medicine practitioners, such as acupuncturists, naturopaths or homeopaths (Adjusted OR: 3·00, 95 % CI: 1·06, 8·48). No significant differences in tobacco use, physical activity, weekly hazardous drinking or consultations with other healthcare professionals (general practitioners, registered nurses, physician specialists or physiotherapists) were observed; all these results are shown in Table 2.

**Table 2.** Plant-based diets association with lifestyle factors, health care utilisation and medication

Lifestyle, use of health care services and medicine consumption	Omnivores		Plant based		Unadjusted OR	Adjusted OR^†^	95 % CI	*P* value
	*n* 342		*n* 86					
	*n*	%	*n*	%				
Self-perceived general health (worse or very worse)	19	5·56 %	5	5·81 %	1·05	1·51	0·44, 4·66	0·48
Tobacco consumption:								
No smoking	216	63·16 %	48	55·81 %				
Smoker	55	16·08 %	19	22·09 %	1·55	1·19	0·58, 2·35	0·63
Ex-smoker	71	20·76 %	19	22·09 %	1·2	1·27	0·64, 2·47	0·49
Weekly hazardous drinking	49	14·33 %	6	6·98 %	0·45	0·49	0·18, 1·16	0·13
WHO-described enhancement of physical activity								
No compliance	98	28·65 %	19	22·09 %				
Aerobic compliance	52	15·20 %	15	17·44 %	1·49	1·91	0·82, 4·47	0·13
Muscle-strengthening compliance	6	1·75 %	2	2·33 %	1·72	1·92	0·23, 11·2	0·49
Muscular or aerobic compliance	186	54·39 %	50	58·14 %	1·39	1·53	0·80, 3·03	0·21
At least one consultation in the last year								
General Practitioner	72	21·05 %	13	15·12 %	0·67	0·68	(0·34, 1·35)	0·272
Physician specialist	32	9·36 %	5	5·81 %	0·6	0·65	(0·23, 1·86)	0·427
Physiotherapist, kinesitherapist, chiropractor, or osteopath	57	16·67 %	15	17·44 %	1·06	0·98	(0·49, 1·97)	0·959
Psychologist, psychotherapist or psychiatrist	20	5·85 %	13	15·12 %	*2·87* ^*^	*3·21*	*(1·38, 7·43)*	*0·007* ^*^
Registered nurse or Nurse Practitioner	46	13·45 %	10	11·63 %	0·85	0·73	(0·33, 1·63)	0·443
Complementary medicine practitioner	11	3·22 %	8	9·30 %	*3·09* ^*^	*3*	*(1·06, 8·48)*	*0·038* ^*^
Consumed medicines in the last two weeks								
Prescribed by a physician	186	54·39 %	54	62·79 %	1·42	1·39	(0·77, 2·53)	0·276
Not prescribed by a physician	60	17·54 %	13	15·12 %	0·84	0·74	(0·37, 1·51)	0·414
Vitamins, minerals and tonics	31	9·06 %	12	13·95 %	1·63	1·85	(0·83, 4·14)	0·132
Naturopathic or homeopathic medicines	9	2·63 %	5	5·81 %	2·28	2·31	(0·65, 8·29)	0·197

* : *P* value < 0.05.

† Adjusted by: sex, region, age, studies, household income and social class.

Plant-based diets were associated with a higher proportion of depressive symptoms according to the PHQ8 questionnaire (Adjusted OR 2·58, 95 % CI: 1·00, 6·65) and an increased risk of stroke (adjusted OR 7·08, 95 % CI: 1·27, 39·46); however, this result showed a small subgroup size and a wide CI. No associations were found between plant-based diets and other examined diseases. Table 3 presents the prevalence risks and unadjusted and adjusted logistic regression results.

**Table 3.** Plant-Based diets association with risk of chronic disease

Self-reported diseases	Omnivores		Plant based		Unadjusted OR	Adjusted OR^†^	95 % CI	*P* value
	*n* 342		*n* 86					
	*n*	%	*n*	%				
Hypertension	54·	15·79 %	8·	9·30 %	0·55	0·55	(0·21, 1·47)	0·233
Allergy	44·	12·87 %	16·	18·60 %	1·55	1·11	(0·54, 2·27)	0·774
Asthma (including allergic asthma)	23·	6·73 %	5·	5·81 %	0·86	0·6	(0·19, 1·88)	0·381
Heart disease	10·	2·92 %	3·	3·49 %	1·2	1·97	(0·44, 8·76)	0·373
*Stroke*	*4·*	*1·17 %*	*4·*	*4·65 %*	*4·12* ^*^	*7·08*	*(1·27, 39·46)*	*0·025* ^*^
Malignant tumors (if any)	5·	1·46 %	2·	2·33 %	1·6	1·57	(0·26, 9·5)	0·622
Diabetes	13·	3·80 %	4·	4·65 %	1·23	1·86	(0·48, 7·18)	0·369
Thyroid problems	11·	3·22 %	4·	4·65 %	1·47	1·67	(0·47, 5·91)	0·429
High cholesterol	39·	11·40 %	3·	3·49 %	0·28^*^	0·29	(0·08, 1·08)	0·064
*Depression symptoms*	*20·*	*5·87 %*	*9·*	*10·59 %*	*1·9*	*2·58*	*(1, 6·65)*	*0·049* ^*^
Migraine or frequent headaches	25·	7·31 %	6·	6·98 %	0·95	1·12	(0·4, 3·12)	0·833
Haemorrhoids	12·	3·51 %	6·	6·98 %	2·06	1·58	(0·5, 4·96)	0·438
Kidney problems	9·	2·63 %	2·	2·33 %	0·88	0·81	(0·15, 4·45)	0·812
Varicose veins in the legs	23·	6·73 %	4·	4·65 %	0·68	0·71	(0·22, 2·36)	0·58
Arthrosis (excluding arthritis)	38·	11·11 %	7·	8·14 %	0·71	0·84	(0·31, 2·29)	0·729
Chronic back pain	55·	16·08 %	16·	18·60 %	1·19	1·56	(0·77, 3·15)	0·215
Osteoporosis	8·	2·34 %	2·	2·33 %	0·99	0·9	(0·16, 5·11)	0·906
Chronic skin problems	16·	4·68 %	6·	6·98 %	1·53	1·57	(0·56, 4·42)	0·389

* : *P* value < 0.05.

† Adjusted by sex, region, age, studies, household income and social class. Diseases are self-reports form the survey item: ‘*Has a physician diagnosed any of the following diseases?*’ for depression symptoms, we used the Patient Health Questionnaire (PHQ-8).

## Discussion

This study found a low prevalence of individuals following a plant-based diet in different regions. Those adhering to plant-based dietary patterns reported a high frequency of intake of bread and cereals, pasta, rice, potatoes, legumes, fruits and vegetables. They were generally younger, female, single, with higher education levels and household incomes. However, when comparing plant-based and omnivorous individuals with similar socio-demographic characteristics and BMI, lifestyle factors did not differ.

The proportion of individuals adhering to plant-based diets was 5·62 per 1000 inhabitants. This is consistent with a previous study that combined data from national and European surveys covering the period from 2001 to 2011 and reported a sample prevalence of 0·2 %^(33)^. In neighboring France, a web-based study reported a 3·4 % prevalence of plant-based diets among its 93 823 participants^(34)^; however, this result might be interpreted cautiously due to the absence of a random sampling. Comparatively, the proportion of vegetarians is estimated at 4 % in the USA and 9 % in Italy, Germany and the United Kingdom^(35)^. These figures, however, are derived from data provided by Western vegetarian societies and may be subject to bias, as individuals often overestimate their adherence to a plant-based diet^(36)^. Therefore, our study provides a reliable, population-based estimate of plant-based diet adherence, confirming that while this dietary pattern remains a minority, its prevalence is likely overestimated in self-reported data.

Regional differences in plant-based adherence in Spain, with the highest frequencies found in the northeast regions of the Mediterranean Sea (Catalonia and Valencia), the central northern regions (Basque Country and Navarre) and the Canary Islands. Conversely, the Southern and central regions had the lowest adherence to plant-based diets. These regional differences may reflect underlying cultural and economic disparities not associated with regional average meat consumption per person, where Navarra had the highest meat consumption. At the same time, Catalonia, Valencia and the Basque Country ranked 8th, 10th and 6th, respectively^(37)^. Regarding Regional Gross Domestic Product per capita for 2020, the Basque Country, Navarra and Catalonia ranked 2nd, 3rd and 4th, while Valencia ranked 12^th^. The low adherence to plant-based diets and observed regional disparities highlight the need for more targeted studies to explore dietary patterns within the population in greater detail.

Individuals following a plant-based diet reported a higher frequency of ‘*never eat*’ responses than omnivores, particularly regarding sweets, sugar-sweetened beverages, fast foods and snacks. In contrast, the highest consumption frequencies among plant-based individuals were observed for bread and cereals, pasta, rice, potatoes, legumes, fruits and vegetables. A systematic review analysing the quality of plant-based diets reported that plant-based diets were of higher quality due to a greater intake of whole grains, total fruits, plant-based proteins and seafood protein, along with a lower intake of refined grains and overall protein foods^(38)^. A study using the Modified Healthy Eating Index also reported that plant-based diet quality scored higher than an omnivore diet in food groups with high-energy density (fat), processed meat and plant-based meat alternatives^(39)^. In our opinion, the dietary intake data obtained from the Spanish EHIS could be used in future research to analyse the quality of the Spanish diet in specific population groups. This approach could allow for adjustments based on lifestyle factors, socio-demographic variables and other covariates that may influence dietary choices and composition.

As anticipated, we implemented a study design to compare individuals following a plant-based diet with omnivores of similar socio-demographic characteristics, and we did not find differences in lifestyle habits (tobacco use and physical activity and hazardous drinking), consultations with general practitioners, registered nurses, physician specialists or physiotherapists. Social determinants of health shape nutritional patterns and influence health status^(40)^. However, studies on the impact of plant-based diets often overlook social determinants of health to assess the health outcomes of plant-based diets accurately. Our study’s results showed that lifestyle factors were similar between the matched samples of participants adhering to plant-based diets and omnivorous diets. However, adherence to a plant-based diet was associated with a higher prevalence risk of depressive symptoms and an increased frequency of visits to mental health professionals. A recent meta-analysis reported that meat consumption was associated with lower depression^(41)^. A study involving a population-based cohort of 90 380 participants in France reported an association between depression and a lacto-ovo-vegetarian diet, with an adjusted OR of 1·44 (95 % CI: 1·08, 1·92), and with a vegan diet, an OR of 1·18 (95 % CI: 0·56, 2·47)^(42)^. These results are comparable to our findings, which showed an adjusted OR of 2·58 (95 % CI: 1·00, 6·65) for the association between depression and plant-based diets. Other studies also found that depression among individuals with plant-based diets, focusing on females or males, was associated with higher rates of depressive disorders^(43,44)^. While most studies, with few exceptions, suggest an association between plant-based diets and mental health issues, it is essential to note that causality cannot be established based on observational studies that do not account for participants’ baseline mental health status. Disentangling why mental health status is associated with adherence to a plant-based diet compared with individuals with similar socio-demographic characteristics would require a different research approach (i.e. a qualitative study), given that an experimental design is not feasible in practice in humans.

We observed an association between the vegetarian diet and self-reported prevalence risk of stroke, with vegetarians showing a significantly higher adjusted OR of 7·08 (95 % CI: 1·27, 39·46) compared with omnivores. This finding aligns with a large cohort study reporting a 17 % increased risk of stroke among vegetarians compared with meat eaters (hazard ratio: 1·17, 95 % CI: 1·00, 1·40), and an hazard ratio of 1·35 for vegans, although the latter was not statistically significant^(45)^. Conversely, other extensive cohort studies and a meta-analysis indicated that vegetarian diets might reduce stroke risk, reporting a hazard ratio of 0·86 (95 % CI: 0·67, 1·11) for stroke among vegetarians^(46–48)^. The risk of stroke is primarily driven by chronic diseases and modifiable factors, such as hypertension, smoking, diet, physical activity and alcohol consumption, which together account for >90 % of the population-attributable risk^(49)^. However, we found no significant associations between plant-based diets and these diseases. Other expected impacts between omnivorous and vegetarian groups on cardiovascular risk factors, such as hypertension or cholesterol, might not have been observed because the sample of vegetarians was relatively young. Studying the impact of dietary patterns in older populations adhering to a vegetarian diet may better capture variations in these risk factors to examine with precision the effect of plant-based diets and increase our understanding of how they affect ischaemic stroke risk and other CVD.

### Practical implications

It should not be presumed that individuals adhering to a vegetarian diet are inherently healthier or experience better mental health status than those following other dietary patterns. Such assumptions must be evaluated within specific health and psychological outcomes, as dietary patterns alone do not necessarily correlate with improved health or increased mental health status. Policymakers and health professionals should prioritise personalised, culturally sensitive, inclusive, and evidence-based dietary guidance rather than promoting specific dietary patterns such as vegetarian, vegan, or omnivorous diets. Furthermore, public health initiatives promoting healthy eating should be accompanied by education on balanced nutrition to prevent potential deficiencies and manage expectations regarding health outcomes.

## Limitations

Participants’ recall biases may influence the study findings. However, because this bias likely affects all participants systematically, the reliability of the results remains intact. In addition, no cases were identified in small regions. Although this may raise concerns regarding prevalence estimates, the questionnaire did not directly ask participants whether they consistently exclude animal products but assessed consumption over a recent reference period. As such, it may not fully capture the true proportion of individuals who follow a strictly vegan or vegetarian diet. While the questioning method could be critiqued, the EHIS data are a valuable and reliable reference for the EU. We could not determine when participants began the plant-based diet or how long they maintained it. However, this limitation also affects the group of omnivores who could have followed plant-based diets in the past. Dietary classification was based on self-reported intake using six frequency categories ranging from ‘once or more times a day’ to ‘never’. Participants who usually consume animal products but abstained during the reference period might be misclassified as vegetarians or vegans. Nevertheless, the use of the ‘never’ category provides a relatively strong criterion, helping to minimise the inclusion of recent or inconsistent adopters in the vegetarian or vegan group.

## Conclusions

The prevalence of plant-based diets remains relatively low. Individuals who adhered to plant-based diets were generally younger, female, single and had higher education levels and household incomes. Self-perceived general health and lifestyle factors, such as tobacco consumption, hazardous drinking and physical activity, were similar to those of individuals following omnivorous diets with similar socio-demographic profiles. These findings highlight the importance of prioritising healthy behaviours over specific dietary patterns when addressing patient concerns regarding dietary patterns and personal well-being.

## Supporting information

Echiburu et al. supplementary material Echiburu et al. supplementary material

## Data Availability

Data described in the manuscript, code book and analytic code will be made publicly and freely available without restriction at https://www.sanidad.gob.es/en/estadEstudios/estadisticas/EncuestaEuropea/Enc_Eur_Salud_en_Esp_2020.htm

## Supplementary material

For supplementary material accompanying this paper visit https://doi.org/10.1017/S1368980025100669
