# Supplementary material for: Impact of plant-based diets and associations with health, lifestyle and healthcare utilisation: a population-based survey study
Source: Public Health Nutr. 2025 Jul 17;28(1):e120. doi: 10.1017/S1368980025100669 (PMC12465083; doi:10.1017/S1368980025100669)
Supplement: Echiburu et al. supplementary material [file S1368980025100669sup001.pdf]

Appendix.

Region	Crude (n)<br>Plant-based<br>(vegetarian or vegan)	n weighted	Prevalence<br>(per 1000)	95% Population CI
**Andalusia**	4	10746	1.52	(0.52 - 4.42)
**Aragon**	1	2070	1.85	(0.26 - 13.08)
**Asturias**	4	3440	3.85	(1.34 - 10.98)
**Balearic Islands**	1	1756	1.72	(0.24 - 12.19)
**Canary Islands**	6	19323	9.98	(4.3 - 22.98)
**Cantabria**	3	1198	2.43	(0.69 - 8.51)
**Castille and Leon**	3	10019	4.86	(1.56 - 15.08)
**Castille La Mancha**	1	2656	1.55	(0.22 - 10.96)
**Catalonia**	15	63354	10.01	(5.56 - 17.97)
**Valencia**	10	38568	9.14	(4.68 - 17.75)
**Extremadura**	1	1305	1.44	(0.2 - 10.19)
**Galicia**	4	6965	3.03	(1 - 9.11)
**Community of Madrid**	13	37448	6.64	(3.7 - 11.88)
**Murcia**	2	2575	2.09	(0.48 - 8.97)
**Navarre**	7	6682	12.23	(4.86 - 30.4)
**Basque Country**	9	13836	7.49	(3.48 - 16.02)
**La Rioja**	2	1266	4.78	(1.03 - 22)
**Ceuta and Melilla**	0	0	0	(0 - 0)

Table A1: Plant-based prevalences.

Region	Crude (n)<br>vegan	n weighted	Prevalence<br>(per 1000)	95% Population CI
**Andalusia**	0	0	0	(0 - 0)
**Aragon**	1	2070	1.85	(0.26 - 13.08)
**Asturias**	2	1150	1.29	(0.32 - 5.14)
**Balearic Islands**	0	0	0	(0 - 0)
**Canary Islands**	1	4687	2.42	(0.34 - 17)
**Cantabria**	1	392	0.79	(0.11 - 5.64)
**Castille and Leon**	1	3505	1.7	(0.24 - 11.99)
**Castille La Mancha**	1	2656	1.55	(0.22 - 10.96)
**Catalonia**	3	17405	2.75	(0.8 - 9.46)
**Valencia**	1	3749	0.89	(0.12 - 6.28)
**Extremadura**	0	0	0	(0 - 0)
**Galicia**	0	0	0	(0 - 0)
**Community of Madrid**	3	8814	1.56	(0.47 - 5.17)
**Murcia**	0	0	0	(0 - 0)
**Navarre**	0	0	0	(0 - 0)
**Basque Country**	4	7440	4.03	(1.34 - 12.08)
**La Rioja**	0	0	0	(0 - 0)
**Ceuta and Melilla**	0	0	0	(0 - 0)

Table A2: Vegan prevalences.

Region	Crude (n) vegetarian	n weighted	Prevalence (per 1000)	95 percent CI
Andalusia	4	10746	1.52	(0.52 - 4.42)
Aragon	0	0	0	(0 - 0)
Asturias	2	2290	2.56	(0.62 - 10.53)
Balearic Islands	1	1756	1.72	(0.24 - 12.19)
Canary Islands	5	14636	7.56	(3.02 - 18.81)
Cantabria	2	806	1.64	(0.33 - 8.11)
Castille and Leon	2	6514	3.16	(0.78 - 12.71)
Castille La Mancha	0	0	0	(0 - 0)
Catalonia	12	45949	7.26	(3.74 - 14.06)
Valencia	9	34820	8.25	(4.05 - 16.71)
Extremadura	1	1305	1.44	(0.2 - 10.19)
Galicia	4	6965	3.03	(1 - 9.11)
Community of Madrid	10	28634	5.08	(2.6 - 9.9)
Murcia	2	2575	2.09	(0.48 - 8.97)
Navarre	7	6682	12.23	(4.86 - 30.4)
Basque Country	5	6396	3.46	(1.21 - 9.83)
La Rioja	2	1266	4.78	(1.03 - 22)
Ceuta and Melilla	0	0	0	(0 - 0)

Table A3: Vegetarian prevalences.

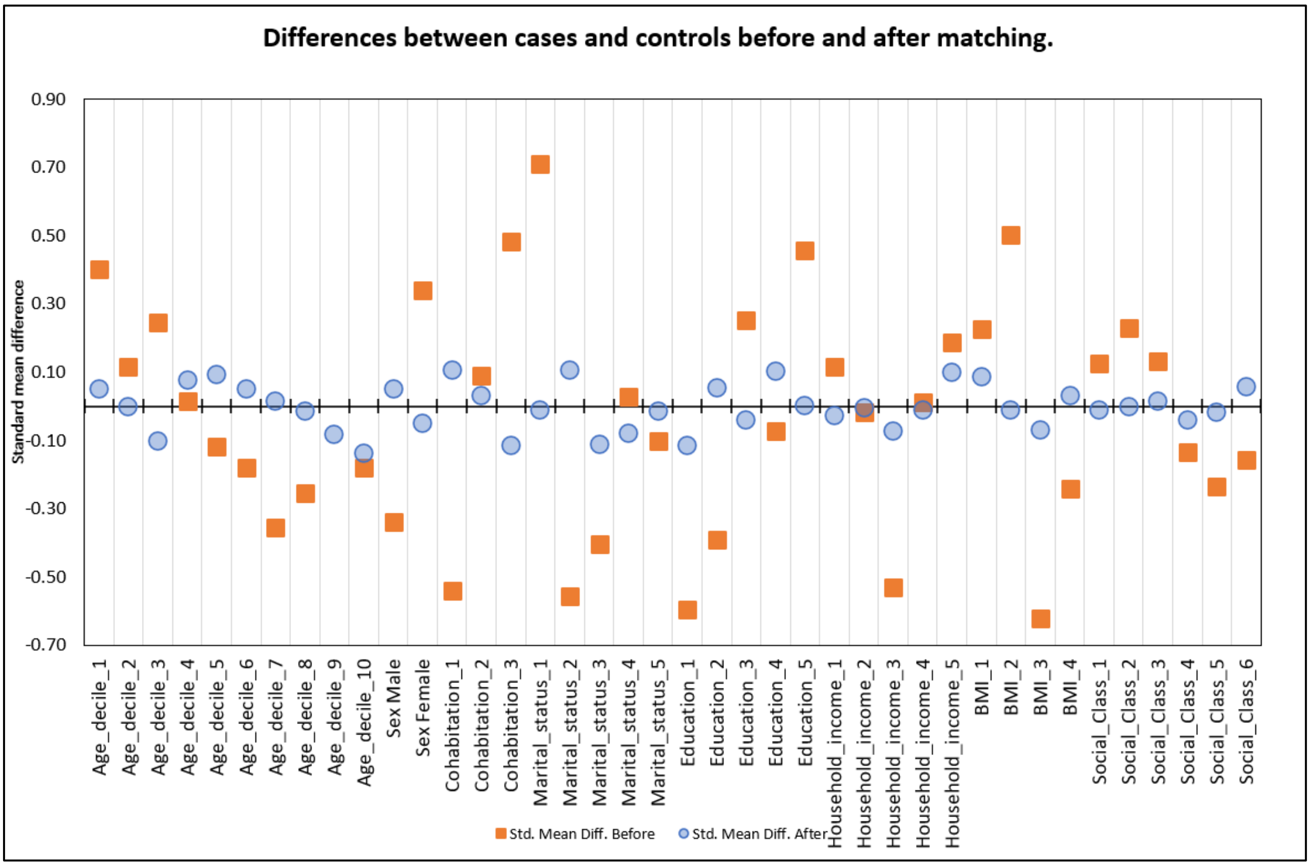

Figure A: Mean standardized differences of selected factors before and after matching.

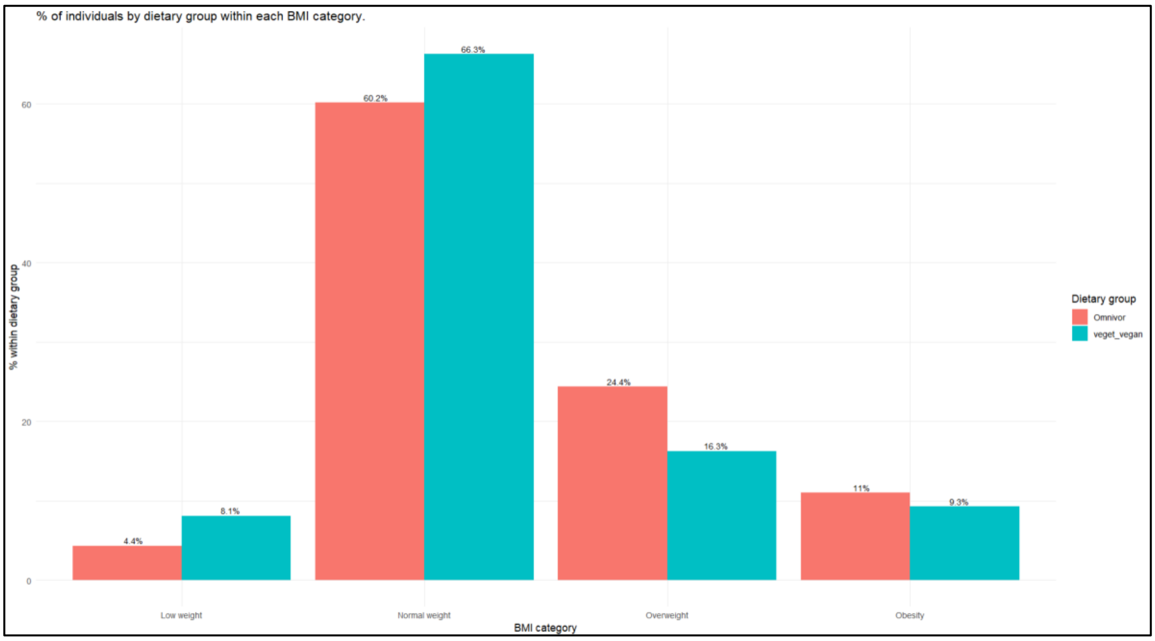

Figure B: Results from a sensitivity analysis assessing BMI differences between omnivorous and vegetarian groups when BMI was not included in the matching process. No statistically significant differences were observed.
